# Nutritional status and anxious and depressive symptoms in anorexia nervosa: a prospective study

**DOI:** 10.1038/s41598-020-79410-y

**Published:** 2021-01-12

**Authors:** A. Pleplé, C. Lalanne, C. Huas, L. Mattar, M. Hanachi, M. F. Flament, I. Carchon, F. Jouen, S. Berthoz, N. Godart

**Affiliations:** 1 Laboratoire Cognitions Humaine et Artificielle, École Pratique des Hautes Etudes, Paris, France; 2 CESP, Univ. Paris-Sud, UVSQ, INSERM U 1178, Université Paris-Saclay, 94805 Villejuif, France; 3 University Paris Diderot, Paris Sorbonne Cité, Paris, France; 4 Fondation Santé des Etudiants de France, Paris, France; 5 Department of Natural Sciences, Lebanese American University, Beirut, Lebanon; 6 UFR des Sciences de la Santé Simone Veil, Université de Versailles Saint-Quentin-en-Yvelines, Versailles, France; 7 Department of Nutrition, Hôpital Raymond Poincaré, Garches, France; 8 University of Ottawa Institute of Mental Health Research, Ottawa, ON Canada; 9 Clinical Nutrition, Geneva University Hospital, Rue Gabrielle-Perret-Gentil 4, 1211 Geneva 14, Switzerland; 10 Département de Psychiatrie, Institut Mutualiste Montsouris, Paris, France; 11 Faculté de Médecine, Université Paris Descartes, Paris, France; 12 CNRS, EPHE, INCIA, UMR 5287, Univ. Bordeaux, 33000 Bordeaux, France

**Keywords:** Nutrition, Body mass index, Nutrition disorders, Psychiatric disorders

## Abstract

The interweaving of malnutrition and symptoms of anxiety and depression in anorexia Nervosa (AN) is mentioned without any consensus regarding the course of anxious-depressive symptoms in relation to nutritional status in the course of treatment of patients with AN. The objectives of the current study in a large sample of AN inpatients were to assess the relationships between anxiety and depression symptoms and nutritional status both over the course of inpatient treatment and at discharge. 222 consecutive inpatients with AN (DSM-IV TR) were assessed (entrance and discharge) for duration of illness, psychiatric treatments, sociodemographic data and with psychometric scales for different psychopathological symptoms [depressive (BDI), anxiety and depressive (HAD scale), obsessive–compulsive (MOCI) and social phobia (LSAS fear score)]. Nutritional status was assessed with Body Mass Index (BMI) and body composition by bioelectrical impedance. The Fat free mass index [FFMI = FFM (kg)/height (m^2^)] was considered for the analysis. Two models were developed where the dependent variables were each psychopathological score at discharge (BDI, HAD anxiety, MOCI, and LSAS fear) in the cross-sectional model, and their variation in the longitudinal model (where a positive score reflected symptom decrease at discharge). A fixed set of predictors, defined on presumed clinical and statistical relevance (FFMI in the cross-sectional model and Variation of FFMI in the longitudinal model), were considered in each model, without any model selection procedure. This is the first study to confirm a positive relationship between the course of eating disorder symptoms and that of anxious-depressive symptoms during inpatient treatment of AN even after adjustment on a vast array of possibly confounding factors.

## Introduction

Anorexia nervosa (AN) is accompanied by both psychiatric and somatic symptoms and characterised by extreme dieting, severe weight loss and resulting malnourishment. Besides its frequent comorbidity with anxiety and depressive disorders^1^, the clinical symptoms of AN commonly include anxious and depressive symptoms^2^. The aetiology of these anxious and depressive symptoms is under discussion : they can be linked either to AN (fear of eating in public, sleep disturbances …) or to the consequences of malnutrition (apathy, eating rituals…) or to psychiatric comorbidities^3–6^.

Keys et al.^7^ were the first to demonstrate a relationship between malnutrition and the presence of anxious-depressive symptoms (i.e. irritability, obsession with food, asthenia, sleep disturbances, and sexual dysfunction). Yet, although the interweaving of malnutrition and symptoms of anxious-depression in AN is often mentioned in the literature, empirical data on this relationship is limited^8,9^ (See Table 1 and Supplementary material S1). After presenting the state-of-the-art, this article aims to provide data on the subject.

**Table 1:** Studies demonstrating a lack of correlation between anxious-depressive symptoms and nutritional status among patients with anorexia nervosa.

	Anxiety			Depression	Comments on the lack of correlation provided by the authors
	Generalized anxiety symptoms	Obsessive–compulsive symptoms	Social phobia symptoms		
BMI	Morgan, Lacey and Reid (1999), Kawai et al. (2008), Sala et al. (2011), Mattar, Huas et al. (2012), Mattar, Thiébaud et al. (2012) and Gauthier et al. (2014)	Mattar, Thiébaud et al. (2012), Mattar, Huas et al. (2012) and Gauthier et al. (2014)	Mattar, Thiébaud et al. (2012), Mattar, Huas et al. (2012), Gauthier et al. (2014), Coulon, Jeammet and Godart (2009)	Mattar, Thiébaud et al. (2012), Mattar, Huas et al. (2012), Gauthier et al. (2014), Kawai et al. (2008), Brockmeyer et al. (2012) and Sala et al. (2011)	The study sample was too homogeneous in terms of malnutrition (Mattar, Huas et al. 2012) The assessment of nutritional status was not good enough, lacking biological or body composition markers (Mattar, Thiébaud et al. 2012; Mattar, Huas et al. 2012; Coulon, Jeammet, and Godart 2009) Comorbidities with depressive and anxiety disorder were not considered (Mattar, Thiébaud et al. 2012; Mattar, Huas et al. 2012; Ricca et al. 2010; Sala et al. 2011; Coulon, Jeammet, and Godart 2009; 2009) Sample size was small (Morgan, Lacey and Reid 1999; Kawai et al. 2008; Ricca et al. 2010; Brockmeyer et al. 2012; Mattar, Thiébaud et al. 2012; Gauthier et al. 2014) Hospitalization and community living increased anxiety (Morgan, Lacey and Reid 1999) Some of the symptoms related to malnutrition disappeared but links to other reasons persisted (Gauthier et al. 2014) Eating symptomatology was the link between nutritional status and anxious-depressive symptoms (Laessle, Schweiger and Pirke 1988; Coulon, Jeammet and Godart 2009; Ricca et al. 2010; Sala et al. 2011; Mattar, Thiébaud et al. 2012) No consideration of confounding factors (Coulon, Jeammet and Godart 2009; Mattar, Thiébaud et al. 2012) Partial renutrition : level and duration of malnutrition were insufficient (Mattar, Thiébaud et al. 2012; Gauthier et al. 2014) Impact of choice and Kinetic characteristics of biological parameters for nutritional status, not all related to anxious-depressive symptoms (Laessle, Schweiger and Pirke 1988) Results depended on the choice of the psychometric scale: a measure assessing somatic symptoms of anxiety was more likely to find a relationship since they are closer to symptoms of malnutrition (Mattar, Thiébaud et al. 2012) Low sensitivity to change of some psychometric measures (Mattar, Thiébaud et al. 2012)
Body weight		Channon and deSilva (1985)		Channon and deSilva (1985), Laessle, Schweiger, and Pirke (1988) (1 of 2 measures)	
Body composition	Mattar, Huas et al. (2012) and Gauthier et al. (2014)			Mattar, Huas et al. (2012)	
Weight loss	Mattar, Huas et al. (2012) and Mattar, Thiébaud et al. (2012)				
Blood albumin				Mattar, Huas et al. (2012)	
Blood BHBA, T3, cortisol				Laessle, Schweiger and Pirke (1988) (1 of 2 measures)	

*BHBA* beta-hydroxybutyric acid.

Since these two above-mentioned reviews, 3 additional studies have been published^10–12^. Among the studies (N = 21 since 1980) that evaluated nutritional status (mostly based on Body Mass Index (BMI) alone) and anxious and depressive symptoms concurrently among patients with AN, 10 studies examined the links between malnutrition and anxious-depressive symptoms at a given time (cross-sectional analyses)^3,10–18^, 8 provided a longitudinal description of their course separately but did not examine whether they were related^19–26^, and 8 assessed their joint evolution^11–14,17,27–29^. For details see Supplementary material S1 literature review.

There is still a lack of consensus regarding the course of anxious-depressive symptoms in relation to nutritional status during treatment of patients with AN, with divergent findings, possibly explained by methodological differences across studies (treatment duration, assessment of nutritional status, and little consideration of confounding factors such as pharmacological treatment, illness duration and psychiatric comorbidities). Further to this, more than half of the studies (11/21 studies) included fewer than 30 patients, and treatment attrition could be as high as 50%^8^. In addition, nutritional status was mainly assessed with BMI, an index that is not sufficiently sensitive and specific to reflect body composition in case of severe malnutrition^9^.

The objectives of the current study on a large sample of AN inpatients were to assess (1) the relationships between symptoms of anxiety and depression and nutritional status at discharge, cross-sectionally and (2) changes in the relationship between symptoms of anxiety and depression and nutritional status over the course of inpatient treatment, using body composition and taking into account potentially confounding factors^8,9,30^ (age, medication at discharge, AN subtype and duration of hospitalization), known to be associated with comorbidities and illness severity.

Alongside this data, and to clarify the link between nutritional status and anxious-depressive symptoms, two hypotheses were tested: (1) that at the end of treatment, the severity of anxious-depressive symptoms would be related to the patient’s nutritional status whatever the causal mechanism; and (2) that the change in anxious-depressive symptoms during hospitalization would be related to the change in the nutritional status*.*

## Methods

### Subjects

**Figure 1:**
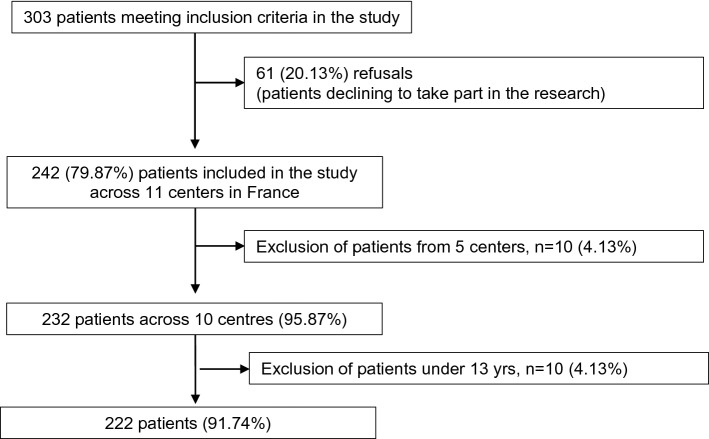
Flow diagram of number of patients included in the present study.

Patients were recruited from the inpatient treatment facilities of 11 centres in France (see EVHAN group for details). Two hundred and forty-two consecutive patients with AN were involved in the EVHAN study from April 2009 to May 2011 (Fig. 1).

Inclusion criteria for the current study were as follows: being hospitalized for AN, admission BMI < 15 and/or sudden and rapid weight loss, agreement to participate in the study, and being affiliated to the French Social Security health coverage system. Exclusion criteria were refusal to participate, insufficient command of the French language, the existence of a potentially confounding pathology (e.g. diabetes, Crohn’s disease or other metabolic disorders) and being under the age of 13. Finally, 222 patients (with full syndrome or sub-threshold AN) were included in this study. A current diagnosis of AN was based on DSM-IV-TR criteria using the Eating Disorder Examination (EDE-Q v. 5.2)^31^ and the CIDI 3.0, with the following criteria for BMI: < 10th percentile up to 17 years of age, and BMI < 17.5 for 17 years of age and older^32^. Participants were assessed in the first two weeks of hospitalization, and again within the two weeks before discharge.

### Ethics statement

This study was part of the larger longitudinal multi-center study *EVHAN* (Evaluation of Hospitalization for AN, Eudract number: 2007-A01110-53, registered in Clinical trials). The study protocol was approved by the Ile-de-France III Ethics Committee and the CNIL (Commission Nationale de l’Informatique et des Libertés). Written informed consent was obtained from each patient before inclusion (adults; minors and their parents). All methods were carried out in accordance with relevant guidelines and regulations.

### Assessment measures

Besides information concerning mental state and nutritional status, socio-demographic data, present weight, minimum and maximum weight with corresponding ages and statures, the last educational level reached, and a global clinical evaluation were collected for the evaluation.

1. Psychopathological evaluations
  Beck’s Depression Inventory 2nd edition (BDI)^33,34^ measures the intensity of depressive symptoms on a 21 items self-report scale. Scores can range from 0 to 63 and index the following categories: up to 11: no depression, 12 to 19: minor depression, 20 to 27 moderate depression, > 27: severe depression. The test retest has been evaluated at r = 0.93^34^.
  The Hospital Anxiety and Depression scale (HAD)^35,36^: a 14 items self-report scale assessing the most frequent anxious-depressive symptoms and their severity. Only the anxiety subscale score was used in the present analyses; it ranges from 0 (no symptoms) to 21 and indexes the following categories: up to 7: no anxiety, 8 to 10: probable anxiety, > 11: caseness of anxiety. Two-weeks test–retest reliability is associated with r > 0.80^37^.
  The Maudsley Obsessive Compulsive Inventory (MOCI)^38,39^: a 30 items self-report scale assessing obsessive–compulsive (OC) behaviors with scores ranging from 0 to 30 (maximum symptoms). A total score of 10 or more has been found to reliably discriminate obsessionals from neurotic patients. One-month test–retest reliability: r = 0.92^40^.
  The Liebowitz Social Anxiety Scale (LSAS)^41,42^: a clinical interview providing a symptom severity score for current fear and avoidance in social interactions (12 items) and performance-oriented situations (12 items). Only the fear subscale was considered in the current study; scores can range from 0 to 72. The threshold score was calculated as above the mean + 2SD (i.e. 20, SD 13.67) endorsed on this subscale by the control participants from the validation study of the LSAS French version^42^.
  The Eating Attitudes Test (EAT-26)^43,44^: a 6-point format, self-report scale measuring a broad range of ED symptoms. It includes 26 items, with 3 subscales: dieting, bulimia and food preoccupations and oral control. Scores range from 0 to 78 (maximum symptoms). A score of 20 or more is considered to reflect clinically significant eating disorders symptoms. The four to five weeks test–retest reliability has been evaluated at r = 0.89^45^.
2. Nutritional status
  *Anthropometry* Body weight and height were measured using standard beam balance scales (Omega-SECA, Germany) and a stadiometer (wall mounted model 222-SECA, Germany) respectively. BMI was derived from weight (kg) divided by the square of height (meters). The severity of weight loss was estimated as the difference between the maximum BMI before illness and the BMI at inclusion in the study.
  *Body composition by bioelectrical impedance (BIA)* Body composition was assessed using the Deurenberg equation^46^, as validated previously on this sample^47^. Fat mass (FM) and Fat free mass (FFM) were adjusted on height to enable an independent evaluation in relation to stature. The FM and FFM indices (FMI and FFMI) were calculated as follows: FFMI = FFM (kg)/height (m^2^) and FMI = FM (kg)/height (m^2^)^48^.
3. Psychotropic medications
  Information on current antidepressants, anxiolytics and antipsychotics at discharge were collected (either introduced or maintained during hospitalization).

### Statistical analyses

Descriptive statistics were first produced for discharge scores (cross-sectional study) as well as for scores assessing changes (variation from admission to discharge: longitudinal). Results are presented as means (and SD). A series of bivariate analyses followed, comparing patients at entrance and discharge (adapted paired tests, Table 2); and comparing patients with or without treatment at discharge (adapted tests, Supplementary Table S1). The relationships between the different psychopathological scores (BDI, HAD anxiety, MOCI, and LSAS fear) and the eating and nutritional status indicators (EAT score at discharge, change in the EAT score in the course of hospitalization, BMI, FFMI, FMI) were tested before and after checking for confounding factors: minimum lifetime BMI, age, duration of illness, duration of hospitalization, restrictive or binging/purging AN and presence/absence of pharmacological treatment (antidepressants, anxiolytics and/or neuroleptics) at discharge, using Pearson’s correlation coefficient (r) or two-tailed Student *t* tests as appropriate. Effect sizes were calculated using Cohen’s d.

**Table 2:** Patients’ (n = 222) characteristics at admission and discharge.

	Admission (A)			Discharge (D)			Variation between (A) and discharge (D)	Paired sample analyses
	N	Mean (SD) Or % (n)	Min–Max	n	Mean (SD) Or % (n)	Min–Max		*p*
AN restrictive subtype	222	47.3(105)	–	–	–	–	–	
Age (years)	222	20.9 (6.6)	13.2–52.3	–	–	–	–	
BMI	222	14.3 (1.5)	10.3–18.9	17.11	17.1 (2.0)	12.6–22.9	2.8 (2.1)	< 0.001
Minimum lifetime BMI	222	13.3 (1.59)	7.61–18.51	–	–	–	–	
Duration of illness (years)	177	3.9 (4.2)	0.2–24.4	–	–	–	–	
Duration of hospitalization (weeks)	–	–	–	204	16.7 (13.5)	1.0–80.0	–	
FFMI at admission	205	12.6 (1.0)	10.4–16.3	143	13.6 (1.3)	11.2–19.6	1.1 (1.1)	*p* < 0.001
FMI at admission	205	1.8 (1.3)	− 1.5–5.1	143	3.5 (1.5)	− 1.9–7.0	1.8 (1.6)	*p* < 0.001
EAT score	219	35.0 (16.7)	0–67.0	168	20.1 (17.1)	0–67.0	− 15.8 (15.0)	*p* < 0.001
EAT score > 19, N (%)		*174 (79.5)*		*168*	*72 (42.9)*			*p* < *0.001*
BDI score	216	26.6 (11.85)	1–53	167	14.32 (10.83)	0–50	− 12.20 (10.59)	*p* < 0.001
BDI categories, N (%)								
No depression		*22 (10.2)*			*81 (48.5)*			*p* < *0.001*
Minor depression		*40 (18.5)*			*39 (23.4)*			
Moderate depression		*57 (26.4)*			*25 (15.0)*			
Major depression		*97 (44.9)*			*22 (13.2)*			
HAD anxiety score	222	12.27 (4.46)	2–21	170	9.20 (4.28)	0–19	− 3.01 (4.37)	*p* < 0.001
HAD anx categories, N (%)								
No anxiety		*36 (16.2)*			*62 (36.5)*			*p* < *0.001*
Probable anxiety		*41 (18.5)*			*36 (21.2)*			
Caseness for anxiety		*145 (65.3)*			*72 (42.4)*			
MOCI score at admission	219	11.56 (5.28)	2–26	167	9.82 (5.07)	1–24	− 1.81 (3.81)	*p* < 0.001
MOCI score > 9, N (%)		*136 (62.1)*			*75 (44.9)*			*0.001*
LSAS fear score	212	31.14 (15.67)	1–71	172	23.24 (16.33)	0–69	− 7.92 (11.54)	*p* < 0.001
LSAS fear score, > 47.34 (%)		*38 (17.9)*			*21 (12.2)*			*0.161*
Treatment	222	–	–	–	–	–	–	
No psychotropic medication		40.1% (89)	–	–	32.4% (72)	–	–	
At least one psychotropic treatment		59.8% (133)	–	–	67.6% (150)	–	–	
Antidepressants^a^		32.9% (73)	–	–	41.4% (92)	–	–	
Anxiolytics^a^		47.3% (105)	–	–	50.0% (111)	–	–	
Antipsychotics^a^		14% (31.0)	–	–	32.4% (72)	–	–	
Hypnotics^a^		17.6% (39)	–	–	8.2% (19)	–	–	
Mood stabilizers^a^		2.3% (5)	–	–	3.2% (7)	–	–	

In italic: proportion of patients with scores above the cut-offs for the corresponding clinical scale (%).

*SD* standard deviation, *BDI* Beck Depression Inventory, *BMI* Body Mass Index, *EAT* Eating Attitude Test, *HAD* Hospital Anxiety and Depression scale, *MOCI* Maudsley Obsessive–Compulsive Inventory, *LSAS* Liebowitz Social Anxiety Scale.

^a^More than one treatment was possible—explaining % above 100.

Paired *t* tests were used to compare scores on the psychopathological measures (BDI, HAD, MOCI, and LSAS) from admission to discharge. As BMI, FFMI and FMI were correlated (BMI-FMI r = 0.784 *p* < 0.001; BMI-FFMI r = 0.574 *p* < 0.001; FFMI-FMI r = − 0.057 *p* < 0.001), FFMI was the only nutritional parameter used in the following analyses.

Finally, two types of multiple linear regression models were run. A fixed set of predictors, defined on presumed clinical and statistical relevance, were considered without any model selection procedure. The adjustment variables were all confounding factors identified by bivariate analyses among the above-mentioned factors.

1. *Cross-sectional models at discharge* Each of the psychopathological scores (BDI, HAD anxiety, MOCI, and LSAS fear) was used as a dependent variable. The independent variables were nutritional status indicators (FFMI) and adjustment variables.
2. *Longitudinal model for evolution in the course of hospitalization* The dependent variables were the variation of the psychopathological scores at discharge (calculated as score at admission minus the score at discharge, centred on the mean; a positive score reflected symptom improvement at discharge) and the explicative variables were changes in FFMI (∆FFMI) as a somatic indicator of nutritional status; and adjustment variables.

*p* values were considered significant at a level of 0.05. Statistical analyses were performed using SPSS 20.0. No adjustments for multiple testing were made^49^.

## Results

### Description

Out of the 222 participants, 105 (47.3%) were of the restrictive-AN type (AN-R) and 117 (52.7%) of the binging-purging-AN type (AN-BP). The clinical characteristics of the participants and global scores on all psychopathological scales at admission and discharge are presented in Table 2. Our sample of AN patients were very severely affected as mean BMI at admission was low (14.3 kg/m^2^), and reached 17.1 at discharge and as the mean duration of EDs was 3.9 years (with a mean age at 20.9). The mean minimum lifetime BMI was 13.3 kg/m^2^.

At admission, the average levels of eating preoccupations and behaviors, anxiety and depression were high (for details see Table 2). At admission, 59.9% were receiving psychotropic medication, and 67.6% at discharge.

Patients with a psychotropic treatment at discharge (as described in Table 2) endorsed higher scores on all the scales than those with no psychotropic medication at discharge. The differences were statistically significant for all the scores except for the LSAS (see Table S1).

### Relationships between psychopathological symptoms and nutritional status indicators

Univariate analyses

1. Cross-sectional analyses at discharge
  At the end of hospitalization, all markers of nutritional status (BMI, FMI and FFMI) were significantly and positively correlated with the level of symptoms of depression (BDI), anxiety (HAD), OCD (MOCI) and social phobia (LSAS) (*p* < 0.05), except for the FMI and LSAS fear scores (social phobia). Correlations between the EAT scores (ED symptoms), and other psychopathological scales (psychological symptoms) were negative (Table 3).
2. Longitudinal analyses (Change scores: evolution in the course of hospitalization).

**Table 3:** Cross-sectional analyses: correlations between nutritional status indicators and psychopathological scores at discharge.

	BDI score	HAD anxiety score	MOCI score	LSAS fear score
BMI				
*r*	− **0.31**	− **0.29**	− **0.22**	− **0.16**
*p*	< 0.001	< 0.001	0.004	0.04
N	167	170	167	172
FFMI				
r	− **0.28**	− **0.28**	− **0.18**	− **0.19**
*p*	0.001	0.001	0.05	0.03
N	131	132	130	131
FMI				
*r*	− **0.27**	− **0.21**	− **0.24**	− 0.12
*p*	0.002	0.01	0.006	0.16
N	131	132	130	131
EAT score				
*r*	**0.61**	**0.57**	**0.32**	**0.40**
*p*	< 0.001	< 0.001	< 0.001	< 0.001
N	165	168	166	155

Bold: Significant associations.

*BDI* Beck’s Depression Inventory, *HAD* Hospital Anxiety and Depression scale, *MOCI* Maudsley Obsessive–Compulsive Inventory, *LSAS* Liebowitz Social Anxiety Scale, *BMI* Body Mass Index, *EAT* Eating Attitude Test, *FFMI* fat-free mass index, *FMI* fat mass index.

From admission to discharge, all markers of nutritional status (BMI, FMI, FFMI) improved significantly (Paired *t* test, *p* < 0.001, Cohen’s d = 1.53, d = 1.27 and d = 0.93 respectively) (Table 4). Similarly, change scores for measures of depressive, anxiety, OC and social phobia symptoms all indicated a significant improvement (Paired *t* test, *p* < 0.001, Cohen’s d = 1.07, d = 0.69, d = 0.35 and d = 0.49 respectively).

**Table 4:** Longitudinal analyses: correlations between change scores for nutritional markers and change scores for psychopathological measures during hospitalization.

	∆BDI score	∆HAD anxiety score	∆MOCI score	∆LSAS fear score
∆BMI				
*r*	− 0.10	− 0.06	− 0.06	− **0.17**
*p*	0.22	0.43	0.44	0.03
N	165	170	165	167
∆FFMI				
*r*	− 0.10	− 0.06	0.01	− **0.25**
*p*	0.29	0.50	0.91	0.01
N	124	125	124	118
∆FMI				
*r*	− **0.20**	− 0.11	− 0.10	− 0.15
*p*	0.03	0.21	0.28	0.12
N	124	125	124	118
∆EAT score				
*r*	**0.42**	**0.32**	**0.17**	**0.26**
*p*	< 0.001	< 0.001	0.03	0.002
N	161	166	163	148

Bold: Significant associations.

*BDI* Beck’s Depression Inventory, *HAD* Hospital Anxiety and Depression scale, *MOCI* Maudsley Obsessive–Compulsive Inventory, *LSAS* Liebowitz Social Anxiety Scale, *BMI* Body Mass Index, *EAT* Eating Attitude Test, *FFMI* fat-free mass index, *FMI* fat mass index, *∆ score* change scores (admission minus discharge).

Changes in BMI and FFMI were negatively associated with changes in symptoms of social phobia (∆LSAS): patients with the greatest increase for BMI and FFMI had the greatest decrease in symptoms of social phobia. Changes in the FMI were negatively correlated with changes in depressive symptoms (∆BDI): patients with the greatest increase for FMI had the greatest decrease in depressive symptoms (Table 4).

3. Potential confounding factors
  - Cross-sectional analyses
    At discharge, the level of ED symptoms (EAT) was significantly and positively correlated with the level of symptoms of depression (BDI), anxiety (HAD), OCD (MOCI) and social phobia (LSAS) (*p* < 0.05, Table 3).
    Age was significantly and positively correlated with symptoms of depression (BDI; *r* = 0.17, *p* = 0.03) and anxiety (HAD; *r* = 0.17, *p* = 0.03). Similarly illness duration was significantly correlated with symptoms of depression (BDI; *r* = 0.179, *p* = 0.038). The older the patients, the longer was the duration of the illness (*r* = 0.68, *p* < 0.001), so that only age was retained as a confounding factor in the multivariate analysis. Patients with AN-BP had higher scores for depressive symptoms (BDI score: 16.3 (11.2) vs 12.1 (10.0), *p* = 0.01, Cohen’s d = 0.04) and anxiety symptoms (HAD score: 10.0 (4.2) vs 8.3 (4.3), *p* = 0.01, Cohen’s d = 0.04) than those with AN-R. Compared to patients receiving no medication, those receiving medication at discharge had higher scores for depressive symptoms (BDI: 16.8 (11.0) vs 8.8 (8.4), *p* < 0.001, Cohen’s d = 0.08), anxiety symptoms (HAD: 10.1 (4.0) vs 7.2 (4.3), *p* < 0.001, Cohen’s d = 0.07) and OCD symptoms (MOCI: 10.5 (5.0) vs 8.3 (5.0), *p* = 0.01, Cohen’s d = 0.04).
  - Longitudinal analyses
    From admission to discharge, the level of ED symptoms (EAT score) improved significantly (Paired *t* test, *p* < 0.001, Cohen’s d = 0.95). The improvement in ED symptoms (∆EAT) was positively and significantly correlated with an improvement in depressive (∆BDI), anxiety (∆HAD), and OCD (∆MOCI) symptoms (Table 4; all *p* < 0.05). The improvement in anxiety symptoms (∆HAD) during hospitalization was smaller among patients with AN-BP than among those with AN-R (− 2.3 (4.2) vs − 3.8 (4.5), *p* = 0.03, Cohen’s d = 0.03). Change scores for depressive symptoms (∆BDI), anxiety symptoms (∆ HAD), OCD symptoms (∆MOCI) and social phobia symptoms (∆LSAS) were not linked to medication at discharge. Minimum lifetime BMI and duration of hospitalization were not correlated with changes in any of the psychopathological scores.

Multivariate analyses

1. Cross-sectional model at discharge (Table 5).

**Table 5:** Multivariate analyses: results.

Explained variables	Explicative variables	*β*	*p*	Model
*Cross-sectional analyses* when medication at discharge is entered in the model				
BDI discharge score	FFMI at discharge	− 0.126	0.082	F(5.125) = 20.937 *p* < 0.001 Adjusted R^2^ = 0.434
	EAT discharge score	**0.538**	**< 0.001**	
	Age at admission	0.071	0.306	
	AN subtype	− 0.081	0.237	
	Medication at** discharge**	**0.145**	**0.047**	
HAD anxiety discharge score	FFMI at discharge	− 0.146	0.060	F(5.126) = 14.833 *p* < 0.001 Adjusted R^2^ = 0.346
	EAT discharge score	**0.439**	**< 0.001**	
	Age at admission	0.054	0.465	
	AN subtype	− 0.119	0.104	
	Medication at discharge	**0.161**	**0.039**	
MOCI discharge score	FFMI at discharge	− 0.071	0.398	F(3.126) = 9.701 *p* < 0.001 Adjusted R^2^ = 0.168
	EAT discharge score	**0.300**	**0.001**	
	Medication at discharge	**0.193**	**0.029**	
LSAS fear discharge score	FFMI at discharge	− 0.114	0.198	F(3.117) = 6.911 *p* < 0.001 Adjusted R^2^ = 0.129
	EAT discharge score	**0.342**	**< 0.001**	
	Medication at discharge	0.030	0.751	
Longitudinal analyses				
BDI discharge score	BDI admission score (centred on the mean)	0.529	< 0.001	F(6.116) = 20.659 *p* < 0.001 Adjusted R^2^ = 0.492
	∆ FFMI	− 0.057	0.410	
	**∆ EAT score**	**0.372**	**< 0.001**	
	Age at admission	0.119	0.077	
	AN subtype	− 0.104	0.119	
	Medication	0.087	0.225	
HAD anxiety discharge score	HAD admission score (centred on the mean)	0.427	< 0.001	F(6.117) = 11.302 *p* < 0.001 Adjusted R^2^ = 0.334
	∆ FFMI	− 0.087	0.271	
	**∆ EAT score**	**0.237**	**0.004**	
	Age at admission	0.065	0.399	
	**AN subtype**	− **0.192**	**0.011**	
	Medication	0.108	0.197	
MOCI discharge score	MOCI admission score (centred on the mean)	0.698	< 0.001	F(4.118) = 35.553 *p* < 0.001 Adjusted R^2^ = 0.531
	∆ FFMI	− 0.033	0.613	
	**∆ EAT score**	**0.136**	**0.041**	
	Medication	0.042	0.539	
LSAS fear discharge score	LSAS admission score (centred on the mean)	0.771	< 0.001	F(4.105) = 43.695 *p* < 0.001 Adjusted R^2^ = 0.610
	∆ FFMI	− 0.075	0.225	
	**∆ EAT score**	**0.140**	**0.030**	
	Medication	0.080	0.199	

Bold: Significant associations.

*BDI* Beck’s Depression Inventory, *HAD* Hospital Anxiety and Depression scale, *MOCI* Maudsley Obsessive–Compulsive Inventory, *LSAS* Liebowitz Social Anxiety Scale, *BMI* Body Mass Index, *EAT* Eating Attitude Test, *AN* anorexia nervosa, *∆* variation of variable (admission minus discharge).

42% of the variance (corresponding to the adjusted R^2^) in depression (BDI score) was explained by our model, where only ED symptoms (EAT score) and medication at discharge were significant. The other models explained 35% of the variance in the anxiety score (HAD anxiety score), and nearly 17% of the variance in OCD symptoms (MOCI score). ED symptoms (EAT score) at discharge explained 13% of the variance in symptoms of social phobia (LSAS fear score)—as it was the only variable that remained significant.

When these analyses were conducted stepwise (introducing each variable successively), FFMI was still significantly linked to BDI score and HAD anxiety score after taking into account EAT score, age and AN subtype, but prior to the introduction of medication at discharge.

2. Longitudinal models in the course of hospitalization (Table 5)
  Improvement in all anxiety and depressive symptoms (measured with HAD and BDI scores) was significantly associated with improvement in ED symptoms (∆EAT). Changes in anxiety symptoms (HAD anxiety score) were also independently associated with AN subtype. None of the changes in anxiety and depression-related symptom scores was linked to nutritional status (FFMI). Taking into account fixed predictors (varying for each score according to the bivariate analysis results—see Table 5) and nutritional somatic markers (∆FFMI), these results can be interpreted as follows during hospitalization, a 1-point decrease:
  in the BDI score was associated with a 0.37-point decrease in the EAT score between admission and discharge (*p* < 0.001). This model was significant and explained 49% of the variance (adjusted R^2^).
  in the HAD anxiety score was associated with a 0.24-point decrease in the EAT score (*p* < 0.01). Patients with AN-BP had significantly higher HAD scores than those with AN-R (*p* = 0.011). The impact of ED symptoms and AN subtype on the HAD anxiety score was significant. The model was significant and explained 33% of the variance.
  in the MOCI score was associated with a 0.136-point decrease in the EAT score (*p* = 0.04). The model was significant and explained 53% of the variance.
  in the LSAS score was associated with a 0.140 decrease in the EAT score (*p* = 0.030, Table 5). The model was significant and explained 61% of the variance.

When these analyses were conducted stepwise (introducing each variable successively), the variation of the FFMI was not significantly linked to anxiety or depressive symptoms at any stage (Results upon request).

## Discussion

The objectives of the current study were to examine the relationships between anxiety and depressive symptoms and nutritional status among patients with anorexia nervosa (AN), taking into account the confounding factors identified in the literature, and using two different approaches: a cross-sectional approach at discharge from inpatient treatment, and a longitudinal approach over the course of treatment. A subsample of participants (*n* = 155) had already been studied at treatment admission^11^. Among published studies, the present study includes the largest sample of participants, and is the only one that has adjusted for confounding factors in longitudinal analyses. Furthermore, the assessment of nutritional status included body composition in addition to BMI.

During hospitalization, ED symptoms (EAT score) and somatic indicators of nutritional status (BMI and body composition: FFMI, FMI), as well as symptoms of depression (BDI), anxiety (HAD), OCD (MOCI) and social phobia (LSAS), improved significantly. Considering meaningful clinically decrease, EAT score > 19 decreased from 79.5 to 42.9%. The percentage of patients with a major depression according to BDI scores decreased from 44.9 to 13.2%; meanwhile the percentage of “no depression” patients (as measured with BDI) increased from 10.2% to nearly half of the sample (48.5%). The percentage of patients with a anxiety caseness according to the MOCI score decreased by 17.2%. No significant clinical improvement was found for the decrease of the LSAS score during hospitalization (*p* = 0.161).

In cross-sectional analyses, at admission^11^ and at discharge from hospitalization (current study), there was a link between somatic indicators of nutritional status (BMI and FMI and FFMI) and anxiety and depressive symptoms, but before psychotropic medication was taken into account. This contrasts with our hypothesis. Indications for psychotropic medication are often linked to anxiety and depressive symptoms. Yet, the patients under psychotropic medication at discharge were more depressed and anxious than those with no such treatment at discharge.

At discharge, the severity of depressive (BDI score), anxiety (HAD score) and OC symptoms (MOCI score) was positively related to both the severity of ED symptoms (EAT score) and the presence of antidepressant, anxiolytic and/or neuroleptic treatment. In addition, the severity of symptoms of social phobia (LSAS score) was positively related to that of ED symptoms (EAT score). Thus, it appears that in the course of hospitalization, patients’ psychological response to nutrition rehabilitation varied, in the sense that those with the most severe anxiety and depressive symptoms at discharge maintained the severity of their ED symptoms, and vice versa, while there was no link at the end of treatment between anxious-depressive symptoms and the somatic indicators of nutritional status. Only 2 studies^16,17^ have shown relationships between depressive symptoms and body weight or BMI, but with conflicting results. These studies did not take psychotropic medications into account. We also showed that when psychotropic medications were not taken into account, FFMI was significantly linked to the intensity of all anxious-depressive symptoms: the lower the FFMI, the greater the anxiety and depressive symptoms. This could be linked to the fact that taking a psychotropic medication was associated with a lower FFMI at discharge (13.48 (1.34) vs 14.06 (0.98); *p* = 0.014).

Patients treated with antidepressant, anxiolytic and/or psychotropic medication scored higher for anxiety and depressive symptoms (BDI, HAD and MOCI) at admission^11^ and at discharge from hospitalization (current study). It is likely that patients treated with medication were those with the most severe symptoms and/or diagnosed with a comorbid depressive or anxiety disorder. Therefore, despite a significant improvement in both pharmacological and untreated subgroups, depressive and anxiety symptoms remained more marked at discharge among patients under medication. Understandably, medication for ED patients with the most severe levels of depression and anxiety symptoms are recommended in current guidelines^50–53^ and discussed in a recent review^54^.

Longitudinal findings indicated that improvement in anxious-depressive symptoms (BDI, HAD, MOCI, and LSAS scores) during hospitalization was positively related to a decrease in ED symptoms (EAT score). The improvement in anxiety symptoms varied according to the AN subtype, with a greater improvement among the AN-R than the AN-BP subtype. However, there was no relationship between improvement in anxious-depressive symptoms (BDI, HAD, MOCI, and LSAS scores) during hospitalization and changes in any of the somatic indicators of nutritional status, with no impact of medication on FFMI improvement.

These results can be interpreted as follows.

1. It is likely that the improvement in anxious-depressive symptoms classically observed during refeeding of patients with AN can be attributed, at least partially, to the benefits of hospitalization. For example, depressive symptoms associated with physical and psychological burnout related to the intensity and chronicity of the illness could be alleviated in the course of hospitalization. Also, the progressive socialization over the course of hospitalization could improve symptoms of anxiety^55^. ED symptoms in themselves, generate depressive and anxiety symptoms, and inpatient care, besides pharmacological treatment, includes therapeutic interventions targeting disturbed eating behaviours^51^. Thus, therapeutic efforts towards a normalization of eating, as well as an improvement in interpersonal relationships and socialization (e.g. eating in public), are likely to contribute to the reduction of anxiety and phobic symptoms.
2. It should be noted that several of the psychopathological measures used in the current study include items relating to eating behaviours, such as appetite changes in the assessment of depressive symptoms (BDI), and eating or drinking in front of others and in public places in the assessment of social phobia (LSAS). Thus, scores on these measures could be impacted by symptoms of AN, and this in turn could bias the relationship between the course of eating behaviours and anxious-depressive symptoms. However, we were able to rule out this hypothesis, as the study results remained unchanged when items related to eating from the BDI and the LSAS scales were excluded. Furthermore, scores on measures of anxiety (HAD) and OCD symptoms (MOCI), which do not include any item relating to eating behaviours, were also strongly related to ED symptoms (EAT score). Therefore, we can conclude that the improvement in ED symptoms during inpatient care was linked to the improvement in anxiety and depressive symptoms, independently from any symptom overlap. Explanatory mechanisms could be numerous.
3. A reduction in anxiety symptoms attributable to nutrition rehabilitation could have been masked by an exacerbation of anxiety symptoms linked to weight gain^8^, which could blur the relationship between anxiety and improvement in the nutritional status.
4. AN subtype had an impact on the course of anxiety symptoms. Patients who had binging and/or vomiting or purging episodes were less likely to have an improvement in their anxiety symptoms compared to those with restrictive AN. This is a novel finding that could be important to take into consideration in future treatment interventions.
5. Inpatient treatment for AN includes antidepressant medication for some patients with comorbidity^56^, and this study demonstrated that medication at discharge (antidepressant, anxiolytic and/or neuroleptic treatment) was not related to the course of anxiety and depressive symptoms during hospitalization. This should not lead to conclude to the inefficacy of pharmacological treatment, as suggested by some authors^57^. Rather, for pharmacologically treated patients, it is almost impossible to say whether any improvement was due to medication or to other components of treatment. The initial severity of anxiety and depressive symptoms among medicated patients was much greater than among those who received no medication, therefore persisting at a higher level of severity throughout treatment. For a better assessment of the impact of medication on the severity of anxiety and depressive symptoms among individuals with AN, analyses should take into account lifetime comorbidities with depressive or anxiety disorders leading to medication prescription. All previous studies on the efficacy of any type of psychotropic medication for the treatment of AN have failed to demonstrate their efficacy in this indication^50–54^. However, no study to date has addressed the efficacy of psychotropic medication for anorexic patients with a comorbid depressive or anxiety disorder. Future research should use randomized trials with and without medication to assess the course of depressive and anxiety disorders among patients with AN and a comorbid affective disorder.

There are limitations to the current study that suggest directions for future research in order to confirm or invalidate any relationship between somatic markers of nutritional status and anxiety and depressive symptomatology in AN. Of note, as inpatient treatment only provides an improvement and not recovery^58^ a significant proportion of patients remained substantially symptomatic at discharge for ED symptoms and/or AD symptoms.The physical status of participants was only assessed by anthropometric parameters and body composition, while preliminary results have indicated that biological markers could be important to consider^11,14^. Also, the intensity and duration of malnutrition, and finally diet composition in past weeks could be used to characterize patients’ nutritional status. In AN, the interweaving of anxiety and depressive symptoms related to malnutrition and those stemming from a comorbid disorder is a complex question. For some patients, an improvement in these symptoms could also be related to the treatment of a comorbid disorder using antidepressant, anxiolytic or neuroleptic treatment. A categorical approach with a lifetime evaluation of comorbidities would be interesting to follow up. Finally, the different findings relative to changes in anxiety could be related to the assessment measures used.

## Conclusion

The study confirmed a positive relationship between the course of ED symptoms and that of anxious-depressive symptoms during inpatient treatment of AN, and this was the case even after adjustment on a vast array of possibly confounding factors. We showed a cross-sectional link between severity of depressive, anxiety, OCD and social phobic symptoms and somatic markers of nutritional status (BMI and body composition: FFMI, FMI) among participants with AN at discharge from inpatient treatment, but not once psychotropic medications were taken into account. Further research is needed taking into account (1) biological markers for a refined assessment of nutritional status and (2) comorbidities with depressive and/or anxiety disorders and how this could influence the recovery.

## Supplementary Information

Supplementary Information.

Supplementary Table.
