# Supplementary material for: Nutritional status and anxious and depressive symptoms in anorexia nervosa: a prospective study
Source: Sci Rep. 2021 Jan 12;11:771. doi: 10.1038/s41598-020-79410-y (PMC7804178; doi:10.1038/s41598-020-79410-y)
Supplement: Supplementary file 1 — Supplementary Information. [file 41598_2020_79410_MOESM1_ESM.docx]

*Supplementary material S1 literature review*

*BMI and psychological symptoms*

Eight (8/21) studies showed a significant increase in BMI and a significant decrease in the severity of depressive symptoms during hospitalization, without testing for a link between the two. However in four of them, there was no change in anxiety symptoms ^20,21,23,25^. Any improvement in anxiety related to nutrition rehabilitation could have been counterbalanced by an increase in anxiety due to weight gain ^8^. Other factors could account for this negative result, such as a short duration of hospitalization (average of 30 days ^20^), the small number of participants (n=20^21^,, n=12^23^, n=16^25^ and n=22^26^) and/or the small change in BMI (from 16.8 to 17.7 ^21^, and from 17.3 to 18.3 ^25^).

Twelve (12/21) studies examined the statistical association between nutritional status and anxious-depressive symptoms (8/21), depression (4/21) or anxiety (1/21).

*Nutritional status and anxiety symptoms*

Eight (8/21) studies ^3,11,13–15,17,18,27^ examined the cross-sectional relationships between anxiety symptoms (i.e. generalized anxiety, obsessive-compulsive disorder (OC), social phobia) and nutritional status (assessed by one or more of the following: BMI, body composition, severity of weight loss, and blood concentration of albumin) and found no relationship between anxiety symptoms and the chosen indicators of nutritional status. Only Mattar *et al.* ^11^ reported a significant negative association with symptoms of social phobia (Table 1).

The 3 studies (3/21) that investigated the longitudinal relationships between anxiety symptoms and nutritional status ^3,27,29^ found no association between the increase in BMI and the reduction in symptoms of generalized anxiety ^3,3,29,29^, OC ^3^ or social phobia ^3,27^.

*Nutritional status and depressive symptoms*

Eleven (11/21) studies examined the cross-sectional relationships between depressive symptoms (i.e. depressive symptoms, cognitive and motivational traits, or mood disturbances) and nutritional status (assessed by one or more of the following: BMI, body weight, body composition, severity of weight loss, serum albumin, beta-hydroxybutyric acid (BHBA), T3 and cortisol). All but two failed to demonstrate any relationship between the severity of depressive symptoms and the chosen indicators of nutritional status. In the study by Laessle *et al*. ^16^, a significant association was found between the score on Van Zerssen’s Depression Scale - but not on Beck’s Depression Inventory (BDI) - and body weight and blood BHBA. In the study by Morgan *et al*. ^17^, there was a positive relationship between depressive symptoms (measured by the HADS scale) and BMI with 11 subjects.

Regarding the studies (5/21) that investigated the longitudinal relationships between depressive symptoms (Raskin’s Mood Scale or BDI) and nutritional status (BMI or body weight) ^3,12,14,28,29^, 3 had positive results and established a link between the decrease in depressive symptoms and an improvement in nutritional status.
