# Supplementary material for: Nutritional status and anxious and depressive symptoms in anorexia nervosa: a prospective study
Source: Sci Rep. 2021 Jan 12;11:771. doi: 10.1038/s41598-020-79410-y (PMC7804178; doi:10.1038/s41598-020-79410-y)
Supplement: Supplementary file 2 — Supplementary Table. [file 41598_2020_79410_MOESM2_ESM.docx]

*Supplementary material Table S1: At discharge, comparison of the patients with or without any medication*

| AT DISCHARGE | No Treatment | Treatment (≥1) | p |
| --- | --- | --- | --- |
|  | 72 | 150 |  |
| BDI (mean (SD)) | 8.8 (8.5) | 16.8 (10.9) | <0.001 |
| HAD anx (mean (SD)) | 7.2 (4.3) | 10.1 (4.0) | <0.001 |
| EAT (mean (SD)) | 11.6 (13.4) | 24.1 (17.3) | <0.001 |
| LSAS (mean (SD)) | 20.0 (15.2) | 24.8 (16.7) | 0.074 |
| MOCI (mean (SD)) | 8.3 (5.0) | 10.5 (5.0) | 0.010 |

BDI: Beck Depression Inventory; EAT: Eating Attitude Test; HAD: Hospital Anxiety and Depression scale; MOCI: Maudsley Obsessive-Compulsive Inventory; LSAS: Liebowitz Social Anxiety Scale
